# Identification of Mutator-Derived lncRNA Signatures of Genomic Instability for Promoting the Clinical Outcome in Hepatocellular Carcinoma

**DOI:** 10.1155/2021/1205029

**Published:** 2021-11-11

**Authors:** Xiaolong Tang, Yandong Miao, Jiangtao Wang, Teng Cai, Lixia Yang, Denghai Mi

**Affiliations:** ^1^The First Clinical Medical College, Lanzhou University, Lanzhou City, Gansu Province, China; ^2^The Second Department of Gastrointestinal Surgery, Affiliated Hospital of North Sichuan Medical College, Nanchong City, Sichuan Province, China; ^3^Gansu Academy of Traditional Chinese Medicine, Lanzhou City, Gansu Province, China

## Abstract

**Background:**

Accumulating evidence proves that long noncoding RNA (lncRNA) plays a crucial role in maintaining genomic instability. However, it is significantly absent from exploring genomic instability-associated lncRNAs and discovering their clinical significance.

**Objective:**

To identify crucial mutator-derived lncRNAs and construct a predictive model for prognosis and genomic instability in hepatocellular carcinoma.

**Methods:**

First, we constructed a mutator hypothesis-derived calculative framework through uniting the lncRNA expression level and somatic mutation number to screen for genomic instability-associated lncRNA in hepatocellular carcinoma. We then selected mutator-derived lncRNA from the genome instability-associated lncRNA by univariate Cox analysis and Lasso regression analysis. Next, we created a prognosis model with the mutator-derived lncRNA signature. Furthermore, we verified the vital role of the model in the prognosis and genomic instability of hepatocellular carcinoma patients. Finally, we examined the potential relationship between the model and the mutation status of TP53.

**Results:**

In this study, we screened 88 genome instability-associated lncRNAs and built a prognosis model with four mutator-derived lncRNAs. Moreover, the model was an independent predictor of prognosis and an accurate indicator of genomic instability in hepatocellular carcinoma. Finally, the model could catch the TP53 mutation status, and the model was a more effective indicator than the mutation status of TP53 for hepatocellular carcinoma patients.

**Conclusion:**

This research adopted a reliable method to analyze the role of lncRNA in genomic instability. Besides, the prognostic model with four mutator-derived lncRNAs is an excellent new indicator of prognosis and genomic instability in hepatocellular carcinoma. In addition, this finding may help clinicians develop therapeutic systems.

## 1. Introduction

Liver cancer is one of the leading causes of cancer death in many countries, accounting for 8.2% of total cancer mortality [1]. More than 90% of all liver cancer cases are hepatocellular carcinomas (HCCs) [2]. Now, tangible advancements have been made in HCC treatment, including chemotherapy and immunotherapy [3]. However, the prognosis of patients with HCC did not increase with the progression of treatment. The median survival of advanced HCC patients is about nine months, and the 5-year overall survival (OS) rate is only 10% [4]. It is known to all that HCC is quite a complicated disease identified by molecular and clinical heterogeneity, as well as its progression and treatment response to treatment [3]. Hence, robust and exact prognostic biomarkers or risk models must be developed to help clinicians formulate therapeutic systems.

Genomic instability often relates to tumor-prone phenotypes and required for the acquisition of tumor-initiating mutations [5]. More importantly, many researchers indicated that genomic instability accumulation refers to patients' tumor progression and prognosis [6, 7]. Even though genomic instability mechanisms have not been wholly discovered, abnormal epigenetics has been reported related to genomic instability [8], indicating molecular signature's capability as a quantitative analysis for genomic instability. In one such event, Bao et al. screened two lncRNAs through The Cancer Genome Atlas (TCGA) database and established an effective prognostic model with a genomic instability signature in breast cancer [9]. Furthermore, Wang et al. formed a miRNA network associated with DNA damage response and identified a 10-miRNA signature related to genome instability and prognosis in ovarian cancer [10]. Many studies have shown that gene instability can promote the occurrence and development of HCC [11, 12]. However, little research has concentrated on forming a prognostic model based on genomic instability genes in HCC.

Numerous studies have demonstrated that long noncoding RNA (lncRNA) is widely expressed in human tissues and plays a crucial role in epigenetic regulation, and it can be used as potential biomarkers in various diseases, including cancer [13]. For example, Zhang et al. reported that lnc-Ip53 regulates the acetylation of p53 protein through a negative feedback loop, thereby regulating the growth and drug chemoresistance in HCC [14]. Li et al. reported that LINC00624 segregates the HDAC6-TRIM28-ZNF354C transcriptional corepressor complex apart from the specific genomic loci as a molecular trick, and it can likely be a remedial target in HCC [15]. Furthermore, accumulating evidence proved that lncRNA plays a vital role in genomic instability. Recently, a study suggested that NORAD, a novel lncRNA, can be activated by DNA damage and interact with NARC1 to maintain genomic instability [16]. Although several reports suggested that lncRNA is associated with genomic instability, genome instability-associated lncRNA and their clinical value in cancer remain primarily uninvestigated.

**Figure 1:**
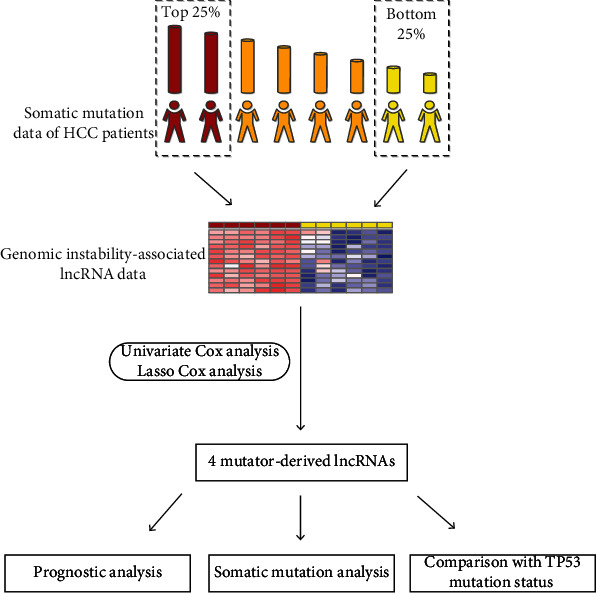
The flow diagram of the study design and analysis.

To research the probability that lncRNA can be an index of genomic instability and prognostic, here, we formed a new prognostic model combining lncRNA expression and somatic mutation. We found that the model constructed by lncRNA is a stable measure to estimate the level of genomic instability and prognosis of HCC patients, and genomic stability had a negative relationship with prognosis in HCC. Moreover, we demonstrated that the model constructed by genomic instability-associated lncRNA is more sensitive than TP53 mutation status to prognosis. TP53 is an important therapeutic target in HCC [17], suggesting that the model constructed by genomic instability-associated lncRNA is a powerful prognostic model and might help clinicians develop therapeutic systems. The flow diagram of the study design and analysis is shown in Figure 1.

## 2. Materials and Methods

### 2.1. Data Sources

Somatic mutation information, RNA-sequencing datasets, and clinical feature of HCC were obtained from the TCGA database (https://portal.gdc.cancer.gov/, v27.0-fix). The matrix files of somatic mutation and RNA-sequencing for different samples were collated and annotated onto the genome through R software (version 4.0.2). Based on the human gene annotation file (http://asia.ensembl.org/index.html), the profiles of lncRNA were acquired from all the RNA-seq datasets.

### 2.2. Bioinformatics Analysis

#### 2.2.1. Screening for Genomic Instability-Associated lncRNA

To screen genomic instability-associated lncRNA in HCC, we constructed a mutator hypothesis-derived calculative framework combing lncRNA expression level and somatic mutation status. First, we count the somatic mutation number of every patient. Second, we sorted patients according to the number of somatic mutations, from highest to lowest. Third, the top 25% and the bottom 25% of the patients are defined as the genomic instability (GI) group and genomic stability (GS) group, respectively. Fourth, we screened differentially expressed instability-associated lncRNA between the two groups using the Wilcox test with R package “limma” [18]. We set the criteria as ∣log fold change∣ greater than 1 and false discovery rate- (FDR-) adjusted *p* value less than 0.05. R package “pheatmap” was used to analyze the instability-associated lncRNA and draw a heatmap [19].

#### 2.2.2. Prognosis Model of Mutator-Derived lncRNA Signature

To further screen the instability-associated lncRNA that correlated with OS, univariate Cox regression analysis and the least absolute shrinkage and selection operator (Lasso) regression analysis were used (*p* < 0.01). With the coefficients from the multivariate regression analysis and the expression level of lncRNA, we calculated the genome instability-derived lncRNA signature (GILncSig) of each patient by the following formula:

(1) $$\begin{array}{c} \text{GILncSig }\left(\text{patient}\right)=\overset{n}{\underset{j=1}{\sum }}\text{c}\text{oef}j*xj \end{array}$$

GILnSig represents the prognostic risk score for HCC patients, coef represents the coefficient, and *xj* represents the expression level of every prognostic mutator-derived lncRNA. We chose the median GILncSig as a cutoff value, and HCC cohorts were separated into the high-risk group and the low-risk group. The Kaplan-Meier (K-M) analysis was employed to assess the survival rate and median survival for each group through R package “survival” and “survminer” [20]. We used the R package “survival ROC” to draw the receiver operating characteristic (ROC) curve for investigating the sensitivity and specificity of the survival prediction by the GILncSig [21]. Area under the curve (AUC) was delivered as an index of prognostic accuracy. Then, we dragged the risk curve and survival state figure together to elucidate the relationship between GILncSig and survival. Through univariate and multivariate Cox regression analyses, we verified whether GILncSig was an independent prognostic factor in HCC.

#### 2.2.3. Survival Analysis According to GILncSig in Different Clinicopathological Features

To further investigate the relationship between GILncSig and clinicopathological features of HCC, the K-M analysis was used based on GILncSig in different groups divided by clinicopathological features.

#### 2.2.4. Exploitation of the Nomogram

It is well known that age, gender, stage, and grade relate to patients' survival with HCC. Thus, we chose age, gender, stage, grade, and GILncSig that we established to perform a nomogram through the R package “rms.” We used calibration traces to check the stability between the real and predicted survival rates. The consistency index (*C*-index), spreading from 0.5 to 1.0, was considered to calculate the model's efficiency for predicting a truthful prognosis. Analyses of 0.5 and 1.0 of the model perform a chance of randomness or outperformance on prognostic survival.

#### 2.2.5. Validation of the Relationship between GILncSig and Genomic Instability

We performed the heatmap, somatic mutation number, and the level of UBQLN4 (a driver of genomic instability [22]) together according to the risk score to better elucidate the relationship between GILncSig and genomic instability. R package “limma” was performed to analyze further the differences in somatic mutation number and level of UBQLN4 between the high-risk and low-risk groups. To more distinctly inspect the variation between the two groups' mutation patterns, we formed a waterfall map through R package “maftools” [23].

#### 2.2.6. Functional Enrichment Analysis of GILncSig Model

To uncover the gene functions and biological pathways of the GILncSig model, we illustrated several significant Gene Ontology (GO) and Kyoto Encyclopedia of Genes and Genomes (KEGG) terms via Gene Set Enrichment Analysis (GSEA) (version 4.1.0) analysis [24–26]. Based on the GILncSig, we separated the patients into two groups. Meanwhile, we organize the expression and annotation profiles as required by GSEA and put them into GSEA. The functional enrichment analysis was shown by R package “plyr,” “ggplot2,” “grid,” and “gridExtra.”

#### 2.2.7. Comparison with TP53 Mutation Status

Since TP53 is a recognized tumor suppressor gene [17], we further compared the effects of GILncSig and TP53 mutation status on survival through the K-M analysis and AUC value of the ROC curve based on the R package “survival,” “survminer,” and “timeROC.” First, we, respectively, examined the effect of risk score on prognosis in the high-risk and low-risk groups. Second, we, respectively, examined the effect of TP53 mutation status on prognosis in the high-risk and low-risk groups. At last, we built the time-dependent ROC curves to further compare the predicted outcome of GILncSig and TP53 mutation status.

## 3. Results

### 3.1. Identification of Genomic Instability-Associated lncRNAs

**Figure 2:**
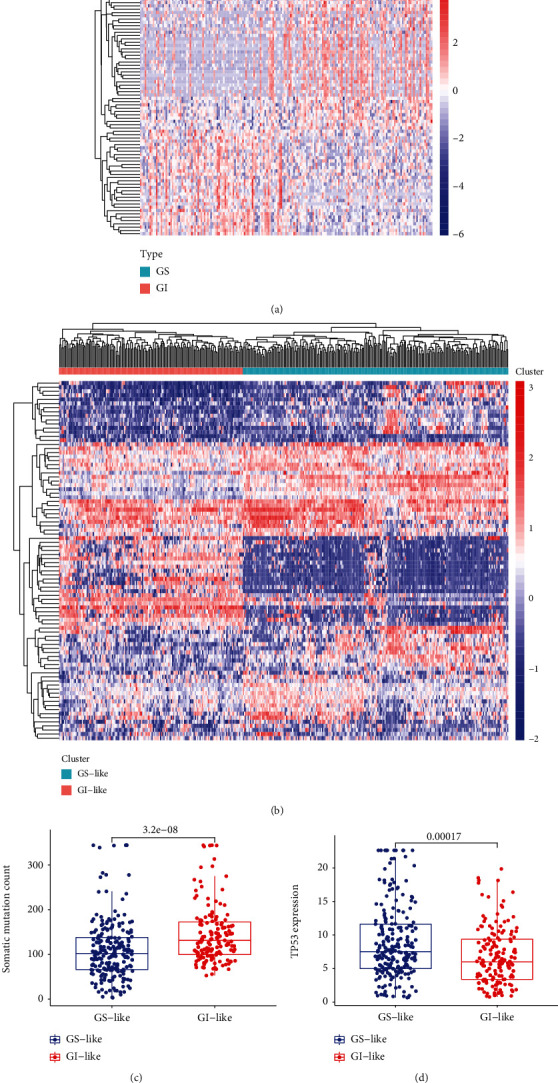
Screening for genome instability-associated lncRNAs (GIlncRNAs). (a) The heatmap of 88 genome instability-associated lncRNAs (GIlncRNAs). The left cyan is the genomic stability (GS) group, and the right red is the genomic instability (GI) group. (b) The unsupervised clustering of HCC patients through the expression model of 88 genome instability-associated lncRNAs. The left red is the genomic instability- (GI-) like group, and the right cyan is the genomic stability- (GS-) like group. (c) The boxplot of somatic mutation in the GS-like and GI-like groups, and the somatic mutation in the GS-like group is significantly lower than the GI-like group. (d) The TP-53 expression level in the GS-like and GI-like groups, and the TP-53 expression level in the GS-like group is significantly higher than the GI-like group.

To screen genomic instability-associated lncRNAs, through sorting the patients by the cumulative number of mutations, we found that 93 patients were in the top 25%, which was the genomic instability (GI) group. 90 patients were in the bottom 25%, which was the genomic stability (GS) group. Based on the SAM method, a total of 88 lncRNAs were identified after we compared the lncRNA level between the two groups (Supplementary Table 1). The lncRNAs contained 32 upregulated and 56 downregulated genes (Figure 2(a)). To confirm whether the function of these lncRNAs relates to genomic instability, we clustered all HCC samples into two groups, the genomic instability- (GI-) like group and genomic stability- (GS-) like group, with 88 differentially expressed lncRNAs by unsupervised hierarchical clustering analysis (Figure 2(b)). The somatic mutation count was significantly higher in the GI-like group clustered as high mutation than the GS-like group clustered as low mutation (*p* = 3.2*e* − 08; Figure 2(c)). Then, we compared the expression level of TP53 between the two groups, and the expression of TP53 in the GI-like group was significantly lower than that in the GS-like group (*p* = 0.00017; Figure 2(d)). These results suggested that the somatic mutation pattern between the GI-like and GS-like groups was significantly different, indicating that these lncRNAs selected above are related to genomic instability. Therefore, we chose these 88 lncRNAs as the candidate for genome instability-associated lncRNAs (GIlncRNAs) for further research.

### 3.2. Prognosis Model of Mutator-Derived lncRNA Signature and Survival Analysis

**Figure 3:**
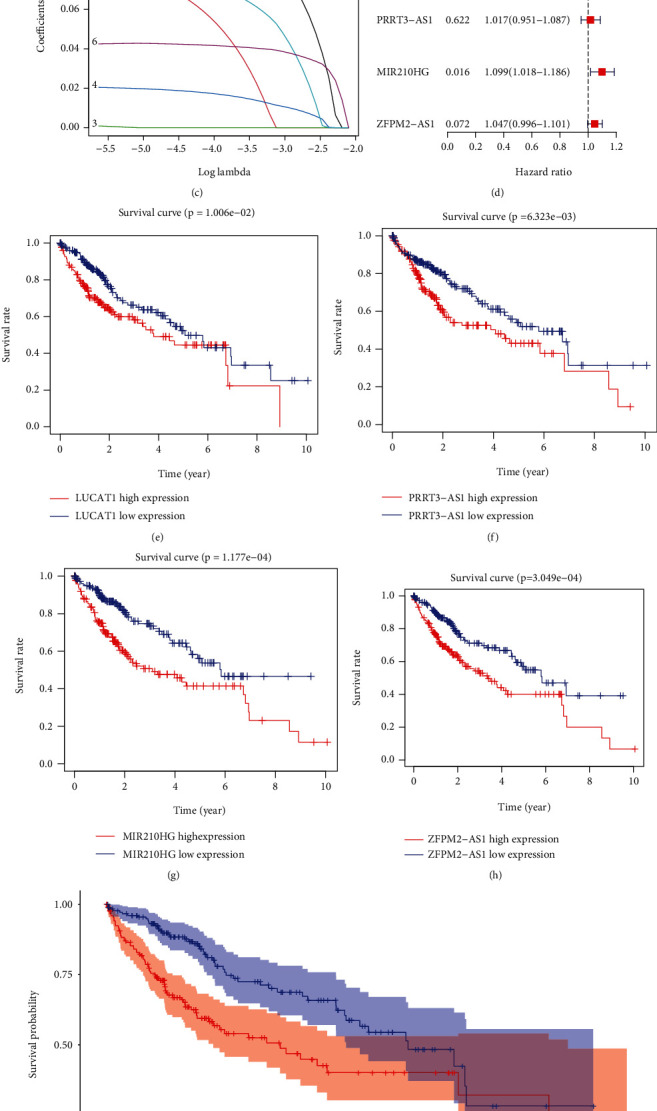
Prognosis model of mutator-derived lncRNA signature. (a) The six prognostic-related GIlncRNAs calculated by univariate Cox regression. (b, c) The process of Lasso regression that we screened out two of the six prognostic-related GIlncRNAs. (d) The four mutator-derived lncRNAs were finally selected and their coefficients. (e–h) The K-M analysis of four mutator-derived lncRNAs. (i) The K-M analysis of HCC patients based on the GILncSig, and the OS of patients in the low-risk group is significantly better than patients in the high-risk group.

**Figure 4:**
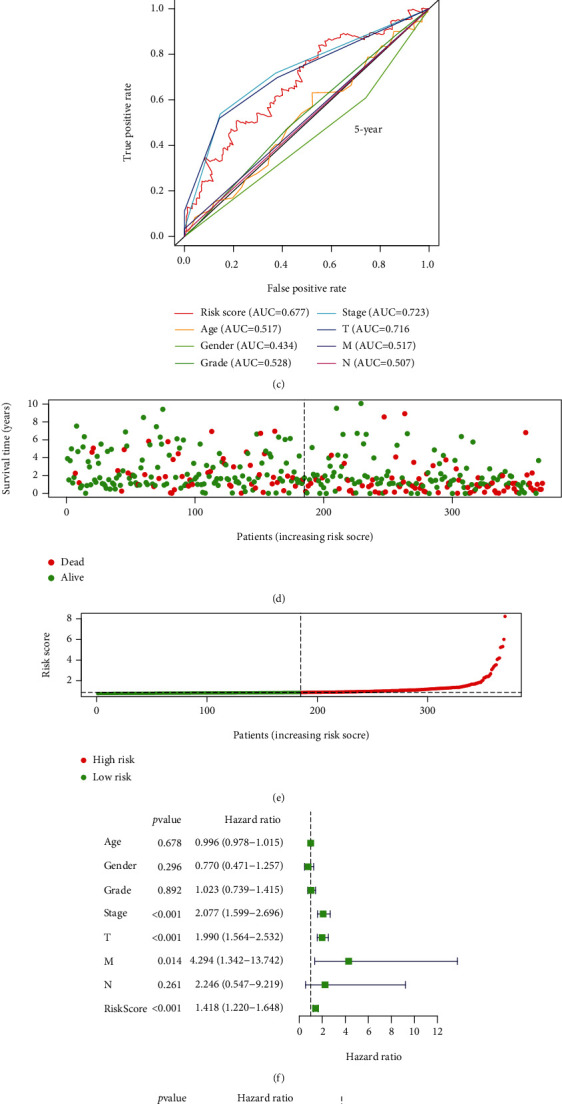
Validation of the relationship between the GILncSig and the prognosis. (a–c) The time-dependent ROC of GILncSig and other clinicopathological features. (d, e) The distribution of the risk score and survival time. The dashed line presents the cutoff value, which divides HCC patients into the low-risk and high-risk groups. Patients' survival time decreased as the GILncSig score increased. (f, g) The GILncSig is an independent prognostic predictor in HCC.

To further filtrate for prognostic-related GIlncRNAs, firstly, we enrolled the univariate Cox regression analysis to assay the relationship between expression levels of GIlncRNAs and OS, and we found six GIlncRNAs (Figure 3(a)). Subsequently, according to Lasso regression results, two GILncRNAs were filtered out (Figures 3(b) and 3(c)). Then, using the multivariate Cox regression analysis, the coefficients of the four GIlncRNAs were calculated (Figure 3(d)). Finally, a mutator-derived lncRNA signature (GILncSig) was established based on the GILncSig expression level and coefficients. The computation of the risk score of GILncSig was shown as follows: risk score = 0.1145 × expression of LUCAT1 + 0.0167 × expression of PRRT3 − AS1 + 0.0942 × expression of MIR210HG + 0.0461 × expression of ZFPM2 − AS1. All 4 GIlncRNAs were high-risk factors due to their HR > 1 (Supplementary Table 2), and the K-M analysis also showed that the expression of the four GIlncRNAs had a significantly negative effect on survival (Figures 3(e)–3(h)). Based on the GILncSig, the K-M analysis demonstrated that the OS of patients in the low-risk group is significantly better than patients in the high-risk group (*p* < 0.001; Figure 3(i)). The time-dependent ROC of GILncSig and other clinicopathological features showed that the AUC of GILncSig of 1, 3, and 5 years were 0.706, 0.682, and 0.677 (Figures 4(a)–4(c)). The distribution of risk scores and survival time suggested that patients' survival time decreased as the GILncSig score increased (Figures 4(d) and 4(e)). Compared with \clinicopathology, such as age, gender, and stage, univariate and multivariate Cox analyses showed that the GILncSig was an independent prognostic predictor in HCC (Figures 4(f) and 4(g)). These results suggested that the GILncSig may be a reliable prognostic indicator in HCC.

### 3.3. Survival Analysis According to GILncSig in Different Clinicopathological Features

**Figure 5:**
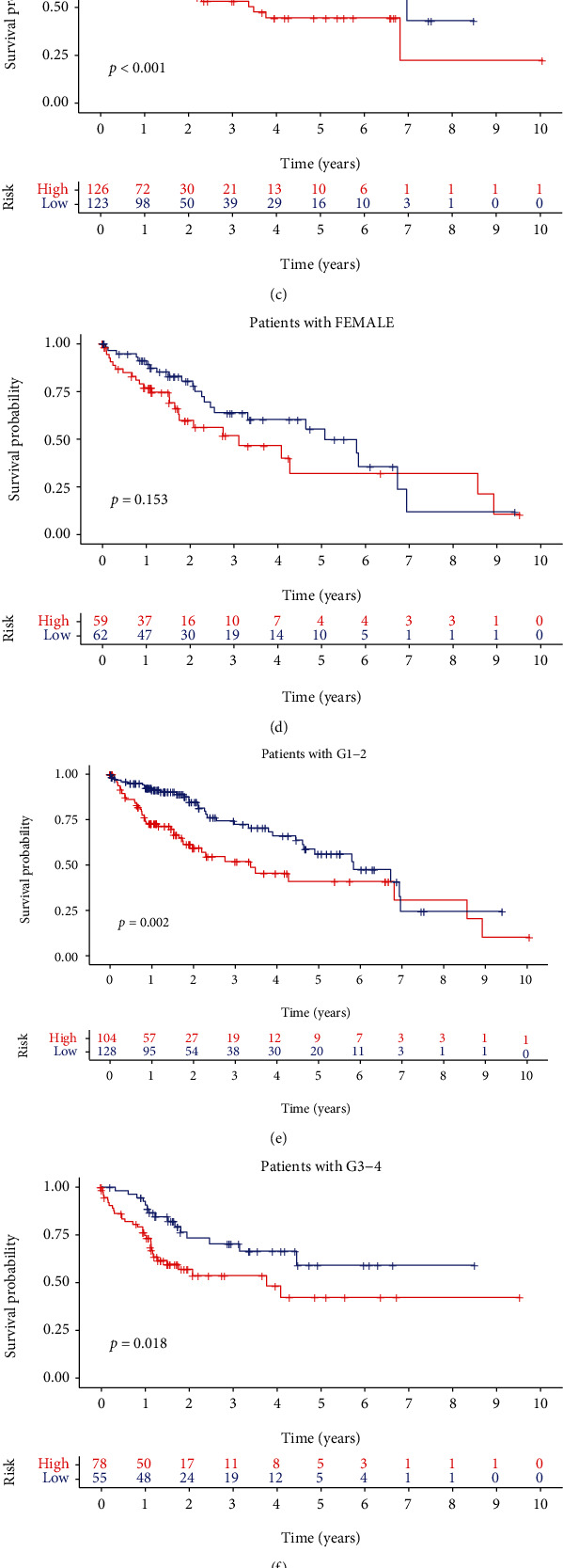
Survival analysis according to GILncSig in different clinicopathological features. (a–h) The K-M analysis according to GILncSig for patients with various clinical features as younger than 65, older than 65, male, female, G1-2, G3-4, T1-2, T3-4. For patients younger than 65, male, G1-2, G3-4, T1-2, T3-4, K-M analysis revealed that the OS in the low-risk group was significantly better than the high-risk group. However, for patients older than 65 or female, there was no significant difference in OS between the high-risk and low-risk groups.

To further investigate the relationship between GILncSig and clinicopathological features of HCC, all patients were classified according to their clinicopathological features and then analyzed for survival based on the GILncSig. For patients younger than 65, K-M survival analysis revealed that the OS of patients in the low-risk group was significantly better than patients in the high-risk group (*p* < 0.001; Figure 5(a)). However, for patients older than 65, there was no significant difference between the high-risk and low-risk groups (*p* = 0.236; Figure 5(b)). For male patients, the OS of patients in the low-risk group was significantly better than patients in the high-risk group (*p* < 0.001; Figure 5(c)). However, no significant difference was found for female patients between the high-risk and low-risk groups (*p* = 0.153; Figure 5(d)). For patients with G1-G2, G3-G4, T1-T2, and T3-T4, K-M survival analysis also showed that the OS of patients in the low-risk group was significantly better than patients in the high-risk group (*p* = 0.002, Figure 5(e); *p* = 0.018, Figure 5(f); *p* = 0.001, Figure 5(g); *p* = 0.04, Figure 5(h)). These results suggested that the GILncSig is a good indicator to predict the prognosis of HCC in males and patients younger than 65, but not in females and patients older than 65.

### 3.4. Exploitation of the Nomogram

**Figure 6:**
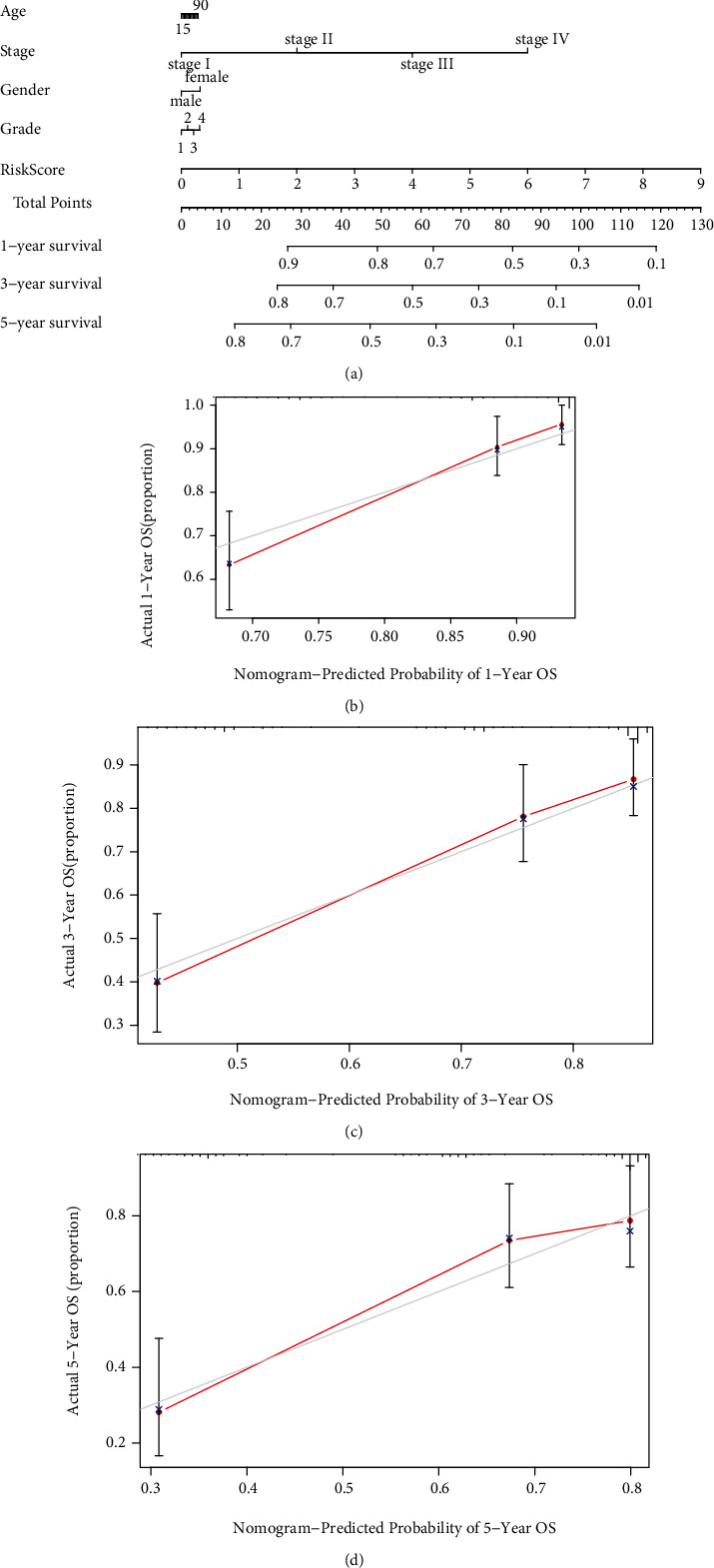
Exploitation of the nomogram. (a) A nomogram for predicting 1-, 3- and 5-year OS based on clinicopathological features and GILncSig in HCC. (b–d) Calibration plots for evaluating the agreement between the predicted and real OS for the prognosis model, and it was used to assess the 1-, 3-, and 5-year OS.

Through merging the age, stage, gender, grade, and risk score, we enrolled a nomogram to predict the possibilities of 1-, 3-, and 5-year OS. Every factor was defined as a score that varies as its donation to survival risk (Figure 6(a)). The calibration curve indicated that the real survival time is consistent with the prognostic survival time, and the *C*-index is 0.736 (Figures 6(b)–6(d)). The results suggested that the nomogram is a reliable and valid method to predict the prognosis of HCC.

### 3.5. Validation of the Relationship between the GILncSig and the Genomic Instability

**Figure 7:**
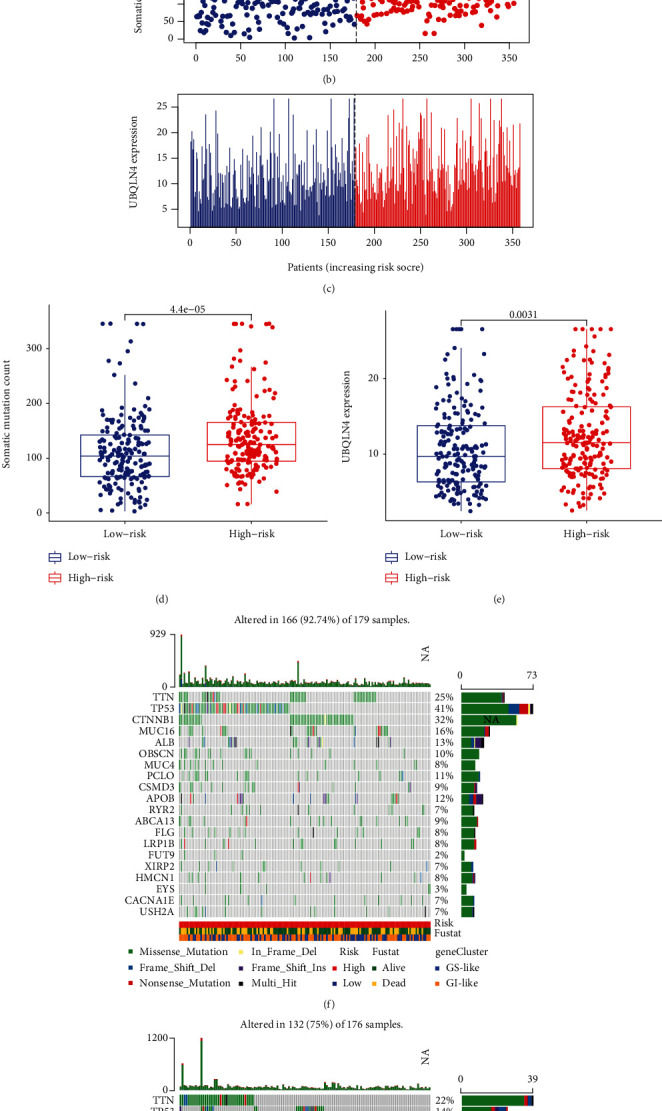
Validation of the relationship between the GILncSig and the genomic instability. (a–c) The distribution of risk score, somatic mutation number, and UBQLN4 expression level. The dashed line presents the cutoff value, which divides HCC patients into the low-risk group and the high-risk group. Patients' somatic mutation number and UBQLN4 expression level increased as the GILncSig score increased. (d, e) The boxplots of somatic mutation number and UBQLN4 expression level. The somatic mutation number and the UBQLN4 expression level in the high-risk group were higher than in the low-risk group. (f, g) The waterfall maps with associated mutation status of the high-risk and low-risk groups. The mutation frequency of TP53 in the high-risk group was 41%, which higher than 14% of the low-risk group.

To validate the relationship between the GILncSig and the genomic instability, we sorted the patients according to their GILncSig score. We found that the number of somatic mutations and the UBQLN4 expression level rise with the growing risk score (Figures 7(a)–7(c)). Since UBQLN4 is a driver gene that can lead to gene instability, we chose it as an indicator of gene instability detection. The comparative analysis results also suggested that the number of somatic mutations and the UBQLN4 expression level in the high-risk group was higher than that in the low-risk group (*p* = 4.4*e* − 05, Figure 7(d); *p* = 0.0031, Figure 7(e)). To more clearly observe the difference between the two groups' mutation patterns, we established a waterfall map (Figures 7(f) and 7(g)). As seen from the waterfall map, the somatic mutation frequency on the whole in the high-risk group was 92.74%, higher than 75% of the low-risk group. Besides, we found that the somatic mutation frequency of TP53 was also significantly different between the two groups. The mutation frequency of TP53 in the high-risk group was 41%, which higher than 14% of the low-risk group. These results suggested significant differences in mutation patterns between the high-risk and low-risk groups, indicating that GILncSig is significantly correlated with genomic instability.

### 3.6. Functional Enrichment Analysis of GILncSig Model

**Figure 8:**
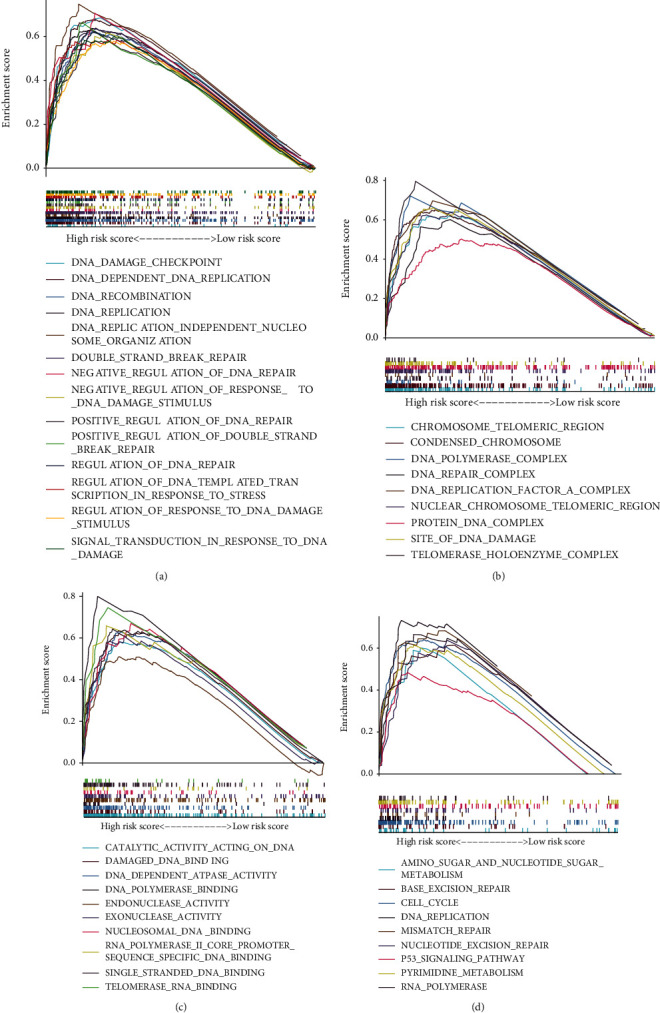
Functional enrichment analysis of GILncSig model. (a) The biological function and processes (BP) enrichment analysis. (b) The cellular component (CC) enrichment analysis. (c) The molecular function (MF) enrichment analysis. (d) The KEGG enrichment analysis.

To uncover the gene functions and biological pathways of the GILncSig model, we illustrated several significant GO and KEGG terms via GSEA analysis (Figures 8(a)–8(d)). The main biological function and processes (BP) include DNA_DAMAGE_CHECKPOINT, DNA_DEPENDENT_DNA_REPLICATION, DNA_RECOMBINATION, and NEGATIVE_REGULATION_OF_DNA_REPAIR. The main cellular component (CC) includes CHROMOSOME_TELOMERIC_REGION, DNA_REPAIR_COMPLEX, PROTEIN_DNA_COMPLEX, and SITE_OF_DNA_DAMAGE. The main molecular function (MF) contains CATALYTIC_ACTIVITY_ACTING_ON_DNA, DAMAGED_DNA_BINDING, DNA_POLYMERASE_BINDING, ENDONUCLEASE_ACTIVITY, and EXONUCLEASE_ACTIVITY (Table 1). The main KEGG enrichment analysis mainly covers the CELL_CYCLE, BASE_EXCISION_REPAIR, DNA_REPLICATION, MISMATCH_REPAIR, NUCLEOTIDE_EXCISION_REPAIR, and P53_SIGNALING_PATHWAY (Table 2). These results included many GO and KEGG terms about gene instability, further ascertaining that GILncSig has a close relationship with genomic instability.

### 3.7. The Predicted Outcome of GILncSig Was Better than That of TP53 Mutation Status

**Figure 9:**
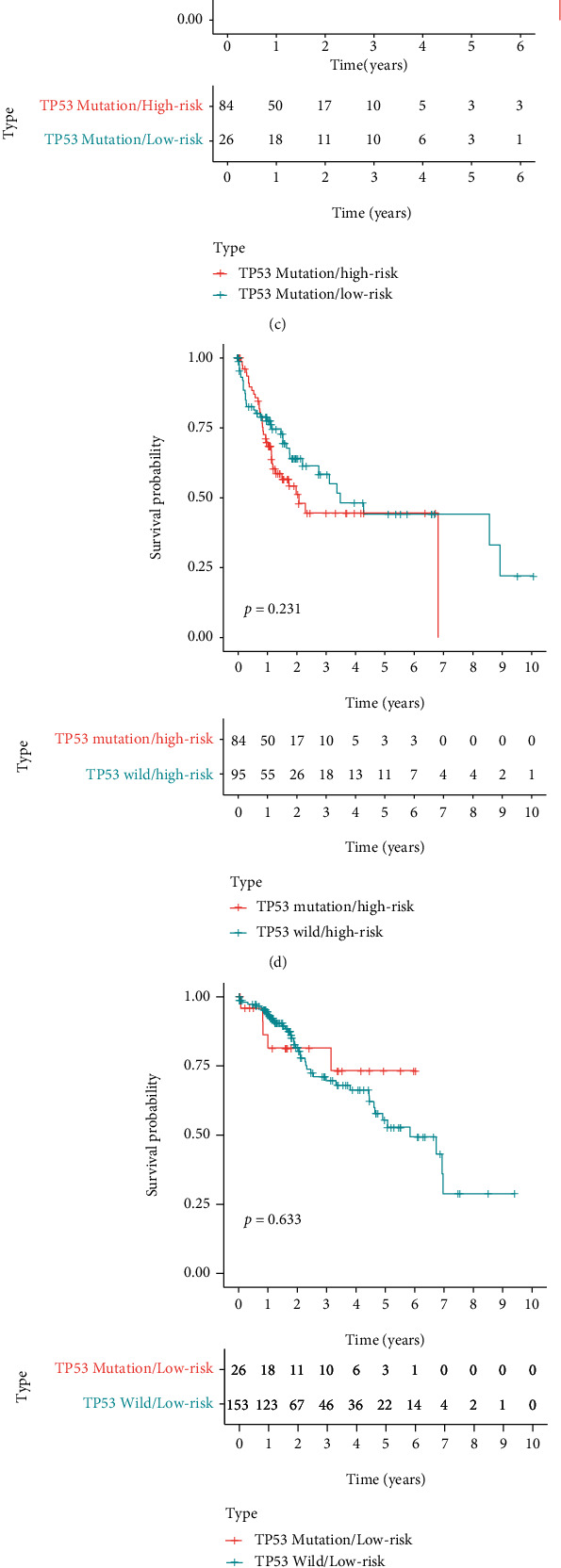
The K-M survival analysis suggested that the predicted outcome of GILncSig was better than that of TP53 mutation status. (a) The K-M analysis of HCC patients with TP53 mutation and TP53 wild, and the OS in the TP53 wild group was significantly higher than the TP53 mutation group. (b) The K-M analysis of the TP53 wild/high-risk group and the TP53 wild/low-risk group, and the OS in the TP53 wild/low-risk group was significantly higher than the TP53 wild/high-risk group. (c) The K-M analysis of the TP53 mutation/high-risk group and TP53 mutation/low-risk group. The TP53 mutation/low-risk group's OS was significantly higher than the TP53 mutation/high-risk group. (d) The K-M analysis of the TP53 mutation/high-risk group and TP53 wild/high-risk group, and there was no significant difference between the two groups. (e) The K-M analysis of TP53 mutation/low-risk group and TP53 wild/low-risk group, and there was no significant difference between the two groups. (f) The K-M analysis of all four groups, and there were significant differences between the four groups.

**Figure 10:**
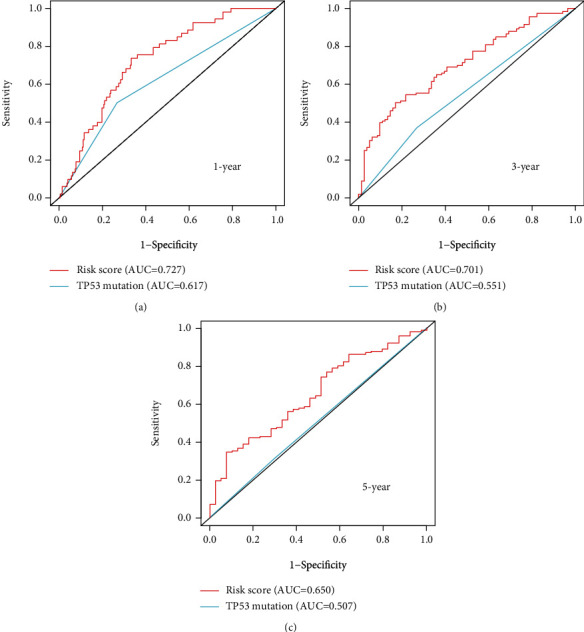
The AUC value of the time-dependent ROC curve suggested that the predicted outcome of GILncSig was better than that of TP53 mutation status. (a–c) The time-dependent ROC of GILncSig and TP53 mutation status in 1 year, 3 years, and 5 years. All AUC values of GILncSig were superior to TP53 mutation status.

We found that somatic mutation patterns of TP53 were significantly different between the high-risk and low-risk groups (Figures 7(f) and 7(g)), suggesting that the GILncSig is related to TP53 mutation status and GILncSig may be a biomarker for TP53 mutation. As we know, TP53 mutation is one of the most common mutations in HCC, affecting the progression and prognosis of HCC. Likewise, we also found a significant difference in survival between patients of HCC with TP53 mutation and without TP53 mutation in TCGA (*p* = 0.013; Figure 9(a)). Hence, we further investigated whether the GILncSig could predict prognosis better than TP53 mutation status. Interestingly, when we applied GILncSig to patients with TP53 wild type and TP53 mutation type, the GILncSig, respectively, divided TP53 wild type and TP53 mutation type patients into two groups with significantly different survival (*p* = 0.016, Figure 9(b), *p* = 0.033, Figure 9(c)). However, when we applied TP53 mutation status to patients with high-risk score and low risk-score, the TP53 mutation status can not divide the high-risk score or low-risk score patients with different survival (*p* = 0.231, Figure 9(d); *p* = 0.633, Figure 9(e)). Moreover, we found that the TP53 mutation/high-risk group's survival curve was more similar to TP53wild/high-risk group but not that similar to the TP53 mutation/low-risk group (Figure 9(f)). In addition, to further verify the predicted outcome of GILncSig and TP53 mutation status, we built the time-dependent ROC curves (Figures 10(a)–10(c)). In 1 year, 3 years, and 5 years, the AUC values of GILncSig were 0.727, 0.701, and 0.650, which were superior to the TP53 mutation status of 0.617, 0.551, and 0.507. Hence, these results suggested that the GILncSig may have a better prognostic value than TP53 mutation status.

## 4. Discussion

In this present study, we constructed a new prognostic model with mutator-derived lncRNAs combining lncRNA expression and somatic mutation, which can accurately evaluate genomic instability and prognosis in HCC. Besides, we demonstrated that the model is more precise than TP53 mutation status on prognosis. Considering TP53 is an important therapeutic target in HCC [17], we suggested that the model built by the mutator-derived lncRNAs is a robust prognostic model and might help clinicians develop therapeutic systems.

88 lncRNAs were found through combing lncRNA expression level and somatic mutation status. Univariate and Lasso Cox analyses selected four mutator-derived lncRNAs from the 88 lncRNAs, including LUCAT1, PRRT3-AS1, MIR210HG, and ZFPM2-AS1. After a detailed review of the literature, we found that these four lncRNAs have been reported related to cancer. LUCAT1 be known as a promoter in pancreatic cancer, non-small-cell lung cancer, and colorectal cancer [27–29]. Fan et al. indicated that PRRT3-AS1 could upregulate migration, proliferation, and invasion of prostate cancer cells through the mTOR pathway [30]. Besides, many recent studies reported that MIR210HG and ZFPM2-AS1 are closely related to cancer progression in various cancers via various ceRNA networks [31–36]. However, the relationship between these four lncRNAs and genomic instability has not been fully discovered yet. These discoveries may offer a basis for further research.

Our model constructed by 4 mutator-derived lncRNAs can accurately predict the prognosis of HCC patients. According to our model, the low-risk group had a longer OS than the high-risk group. It is consistent with previous similar studies that the model was built by lncRNAs in HCC [37, 38]. In addition, the 1-, 3- and 5-year AUC values of our model were more accurate than the model of Shen et al. built by N6-methyladenosine- (m6A-) mediated messenger RNA signatures [39]. Both univariate and multivariate Cox analyses affirmed that the signature could be an independent prognostic indicator compared with other critical clinicopathologic features. These results indicated that GILncSig had a certain potential in predicting survival.

We analyzed the GILncSig in different clinicopathological characteristics groups. The results showed that the GILncSig is an excellent indicator to predict the prognosis of HCC in males and patients younger than 65. However, it is somewhat surprising that GILncSig did not perform the predictive function we expected in females or patients older than 65. This different result in gender may be partly explained by the fact that sex hormones have a crucial role in the development of HCC [40, 41]. Recently, Petrick et al. indicated that a doubling in the concentration of 4-androstenedione (4-dione), a hormone secreted by women during ovulation, was associated with a 50% decreased HCC risk [42]. Moreover, Wei et al. reported that 17*β*-estradiol (E2) could interact with inflammasome and may work as an inhibitor in HCC progression since it triggers pyroptotic cell death and suppresses protective autophagy [43]. Although the mechanism by which sex hormones contribute to the development of HCC remains unclear, it may explain our different results in gender. The different results in age may be somewhat limited by the mechanism that the burden of several DNA damage classes is greater in older than in younger [44]. There are, however, other possible explanations such as sample size, loss to follow-up, or other causes, which need further exploration.

We built a nomogram, a reliable tool, to predict prognosis to quantify individual risk by merging and calculating different risk factors [45, 46]. The most compelling finding is that the risk score and stage contributed a large proportion of the total point from the nomogram. However, age, gender, and grade contributed a tiny proportion to the total point. This result is consistent with the univariate and multivariate Cox analyses and conforms to the HCC's essential characteristics [2].

After a variety of indicators detection, the results indicated that there is a close relationship between our model and genomic instability. The tumor mutator phenotype and UBQLN4 expression level, critical indicators of genomic instability, are significantly correlated with the GILncSig. UBQLN4 is a novel driver gene of genomic instability in cancer. Recently, Jachimowicz et al. indicated that UBQLN4 shortens homologous recombination-mediated DSB repair (HRR) activity by erasing MRE11 from damaged chromatin and contributing to genomic instability [22].

The GO analysis showed that the primary BP of the GILncSig contains the DNA damage checkpoint, DNA-dependent DNA replication, DNA recombination, and negative regulation of DNA repair. This finding is consistent with the progress of genomic instability [47]. The main CC mainly includes chromosome telomeric region, DNA repair complex, protein DNA complex, and site of DNA damage. And the MF mainly contains catalytic activity acting on DNA, damaged DNA binding, DNA polymerase binding, endonuclease activity, and exonuclease activity. The results of CC and MF were also coherent with genomic instability. Thus, it could conceivably be hypothesized that the functional enrichment results of GILncSig were significantly related to genomic instability. The KEGG pathway analysis of the GILncSig mainly includes cell cycle, DNA replication, mismatch repair, base excision repair, and nucleotide excision repair. Surprisingly, the p53 signaling pathway was found in the results, which seems consistent with recent reports that p53 is crucial in maintaining genomic stability [48]. Moreover, Nakajima et al. indicated that p53 mutation could induce genetic instability and aggressive behavior in HCC [49]. Therefore, it was evident that GILncSig has a close relationship with genomic instability and cancer, suggesting that GILncSig is a credible model in predicting genomic instability and prognosis of HCC.

Our results showed that according to GILncSig, the proportion of TP53 mutations was significantly higher in the high-risk group than in the low-risk group, indicating that the GILncSig could catch the TP53 mutation status. Moreover, our results suggested that GILncSig has a better prognostic value than TP53 mutation status in HCC. These results were similar to those of Siqi et al., who also found that the model built by lncRNAs could hold TP53 mutation status and have a greater prognostic significance than TP53 in breast cancer [9]. Hence, this finding further illustrates lncRNA's critical role in maintaining genomic instability and the urgency of exploring lncRNA's mechanism in epigenetics [50].

Some weaknesses need to be noted regarding the present study. A limitation of this study is that the results may have a particular deviation since the number of patients in this analysis is not large. Another vulnerability source assumes that we need more independent datasets to confirm the GILncSig to demonstrate its robustness and reproducibility. Moreover, we should manage more functional experiments to indicate the potential molecular mechanisms for predicting the effect of genomic instability-associated lncRNAs.

## 5. Conclusions

We constructed a mutator hypothesis-derived calculative framework to screen genomic instability-associated lncRNAs. It contributes a vital means for further researching the relationship between lncRNA and genomic instability. Four genomic instability-associated lncRNAs were identified as mutator-derived lncRNAs for the survival of HCC patients. The mutator-derived lncRNA signature was a significant independent factor compared with other important clinical features in HCC. Therefore, the four mutator-derived lncRNAs and their signature might be molecular biomarkers of prognosis. Moreover, they may have important implications for genomic instability and even have the potential to help clinicians develop therapeutic systems in HCC.

## Figures and Tables

**Table 1:** The main GO terms of GILncSig model.

GO	Terms	NES	NOM *p*-val	FDR *q*-val
BP	DNA_DAMAGE_CHECKPOINT	1.81	0.004	0.028
	DNA_DEPENDENT_DNA_REPLICATION	1.87	0.002	0.023
	DNA_RECOMBINATION	1.93	0.002	0.020
	NEGATIVE_REGULATION_OF_DNA_REPAIR	1.92	≤0.001	0.019
CC	CHROMOSOME_TELOMERIC_REGION	1.85	≤0.001	0.021
	DNA_REPAIR_COMPLEX	1.66	0.014	0.049
	PROTEIN_DNA_COMPLEX	1.68	0.008	0.046
	SITE_OF_DNA_DAMAGE	1.79	0.004	0.029
MF	CATALYTIC_ACTIVITY_ACTING_ON_DNA	1.75	0.012	0.046
	DAMAGED_DNA_BINDING	1.69	0.008	0.057
	DNA_POLYMERASE_BINDING	1.86	≤0.001	0.030
	ENDONUCLEASE_ACTIVITY	1.73	0.002	0.046
	EXONUCLEASE_ACTIVITY	1.66	0.006	0.061

**Table 2:** The main KEGG terms of GILncSig model.

KEGG terms	NES	NOM *p*-val	FDR *q*-val
CELL_CYCLE	1.81	0.002	0.048
BASE_EXCISION_REPAIR	1.67	0.014	0.097
DNA_REPLICATION	1.67	0.014	0.093
MISMATCH_REPAIR	1.63	0.030	0.107
NUCLEOTIDE_EXCISION_REPAIR	1.74	0.004	0.080
P53_SIGNALING_PATHWAY	1.60	0.023	0.114

## Data Availability

RNA-seq data, somatic mutation data, and clinical data were collected from the TCGA database.

## Supplementary Materials

Supplementary 1 Supplementary Table 1: genomic instability-associated lncRNAs in HCC.

Supplementary 2 Supplementary Table 2: mutator-derived lncRNAs and prognosis model coefficient.
