# Supplementary material for: Identification of Mutator-Derived lncRNA Signatures of Genomic Instability for Promoting the Clinical Outcome in Hepatocellular Carcinoma
Source: Comput Math Methods Med. 2021 Nov 11;2021:1205029. doi: 10.1155/2021/1205029 (PMC8613502; doi:10.1155/2021/1205029)
Supplement: Supplementary 2 — Supplementary Table 2: mutator-derived lncRNAs and prognosis model coefficient. [file 1205029.f2.docx]

Supplementary Table 2. Mutator-derived lncRNAs and prognosis model coefficient

| id | *coef* | HR | HR.95L | HR.95H | *p*-val |
| --- | --- | --- | --- | --- | --- |
| LUCAT1 | 0.114466324 | 1.121274881 | 1.015804324 | 1.237696404 | 0.023142197 |
| PRRT3-AS1 | 0.016731101 | 1.016871849 | 0.95147151 | 1.086767546 | 0.621806681 |
| MIR210HG | 0.094221705 | 1.098803329 | 1.017725512 | 1.186340268 | 0.01598613 |
| ZFPM2-AS1 | 0.046076177 | 1.047154177 | 0.995923824 | 1.101019821 | 0.071802684 |
